# (2,5-Di­methyl­imidazole){*N*,*N*′,*N*′′,*N*′′′-[porphyrin-5,10,15,20-tetra­yltetra­(2,1-phenyl­ene)]tetra­kis(pyridine-3-carboxamide)}manganese(II) chloro­benzene disolvate

**DOI:** 10.1107/S2414314624004978

**Published:** 2024-06-04

**Authors:** Jun Yang, Cuijuan Zhang, Jiaxiang Chu

**Affiliations:** a School of Chemical Sciences University of Chinese Academy of Sciences, 101408 Beijing People’s Republic of China; University of Aberdeen, United Kingdom

**Keywords:** crystal structure, porphyrin derivative, hydrogen bonds

## Abstract

In the title compound, [Mn(C_68_H_44_N_12_O_4_)(C_5_H_8_N_2_)]·2C_6_H_5_Cl, the central Mn^II^ ion is coordinated by four pyrrole N atoms of the porphyrin core in the basal sites and one N atom of the 2,5-di­methyl­imidazole ligand in the apical site. Two chloro­benzene solvent mol­ecules are also present in the asymmetric unit. Due to the apical imidazole ligand, the Mn atom is displaced out of the 24-atom porphyrin mean plane by 0.66 Å. The average Mn—N_p_ (p = porphyrin) bond length is 2.143 (8) Å, and the axial Mn—N_Im_ (Im = 2,5-di­methyl­imidazole) bond length is 2.171 (8) Å. The structure displays inter­molecular and intra­molecular N—H⋯O, N—H⋯N, C—H⋯O and C—H⋯N hydrogen bonding. The crystal studied was refined as a two-component inversion twin.

## Abstract 2

In the title compound, the central Mn^II^ ion is coordinated by four pyrrole N atoms of the porphyrin core in the basal sites and one N atom of the 2,5-di­methyl­imidazole ligand in the apical site. Two chloro­benzene solvent mol­ecules are also present in the asymmetric unit.

## Graphical abstract

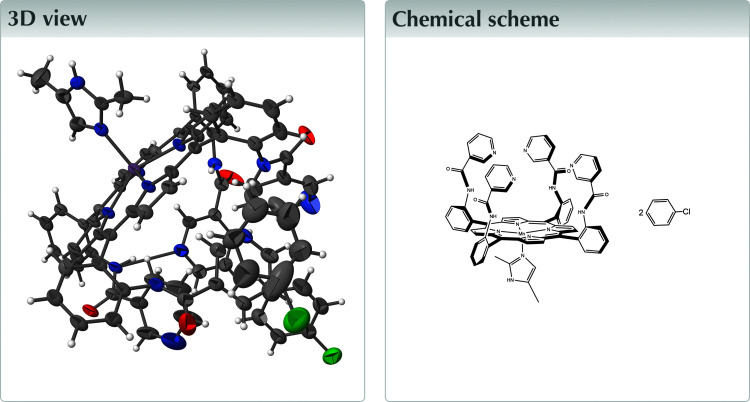

## Structure description

Metalloporphyrins combined with imidazole­(ate) ligands have long been utilized to replicate metalloenzymes, specifically five-coordinate heme complexes (Liang *et al.*, 2023 ▸; Yu *et al.*, 2015 ▸; Yao *et al.*, 2019 ▸; Krishna Deepak & Sankararamakrishnan, 2016 ▸). Imidazole and imidazolates have been extensively employed as axial ligands to imitate histidine residues, which also possess a five-membered ring and play significant roles in the properties and functions of hemoproteins (Nappa *et al.*, 1977 ▸). The first imidazole manganese porphyrin adduct, [Mn(TPP)(1-MeIm)], (TPP = 5,10,15,20-tetra­phenyl­porphyrin, 1-MeIm = 1-methyl­imidazole) was documented by Scheidt and colleagues in 1977 (Kirner *et al.*, 1977 ▸). Subsequently, in 1980, Reed and coworkers reported the first imidazolate manganese porphyrin adduct (Landrum *et al.*, 1980 ▸). In this study, the synthesis and crystal structure of the title manganese(II) porphyrin solvated complex, [Mn(C_68_H_44_N_12_O_4_)(C_5_H_8_N_2_)]·2C_6_H_5_Cl, is presented.

**Figure 1:**
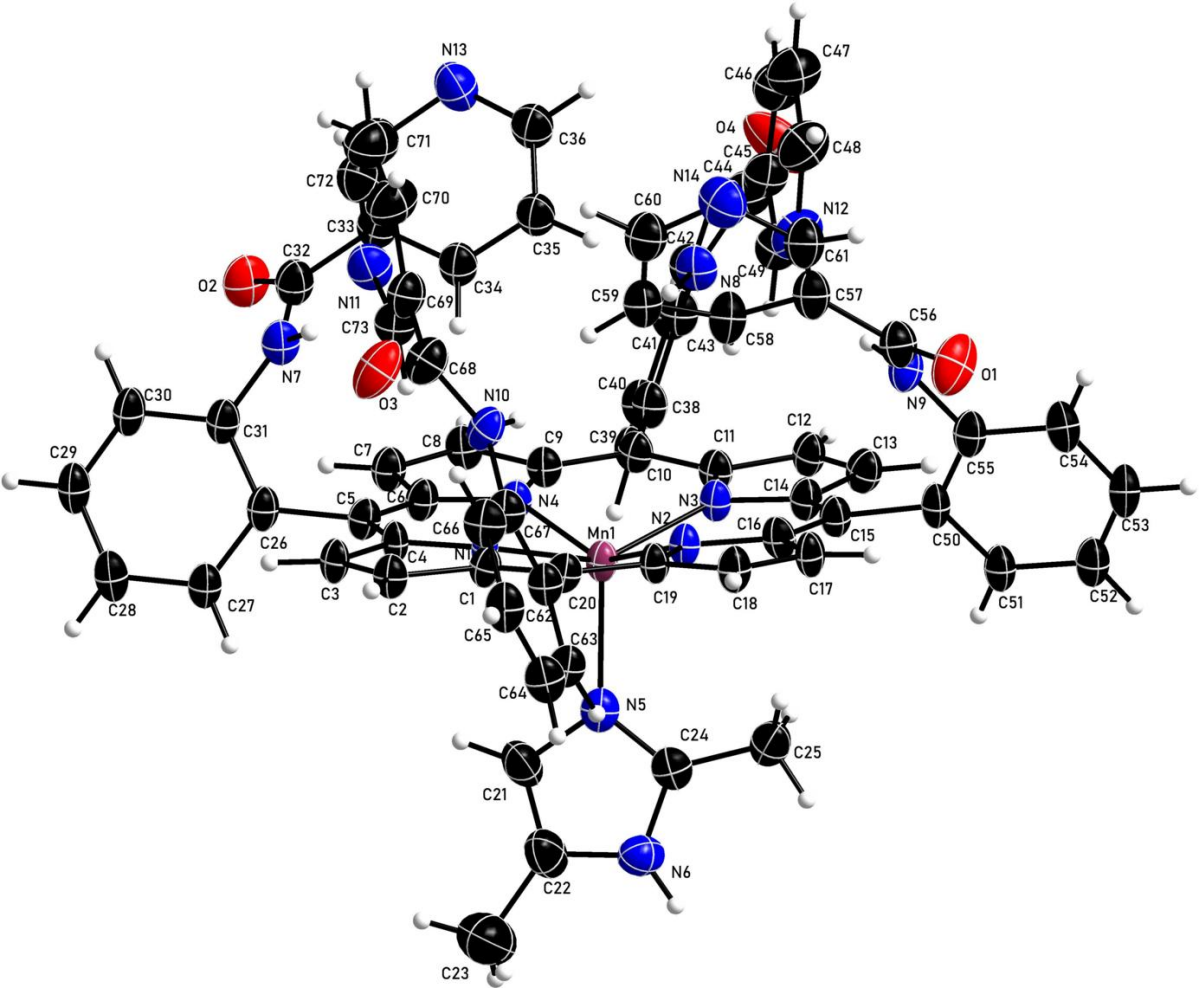
The mol­ecular structure of the title compound with displacement ellipsoids drawn at the 50% probability level. The solvent mol­ecules have been omitted for clarity.

The asymmetric unit of the title compound contains one (2,5-di­methyl­imidazole){*N*,*N*′,*N*′′,*N*′′′-[porphyrin-5,10,15,20-tetra­yltetra­(2,1-phenyl­ene)]tetra­kis­(pyridine-3-carboxamide)}manganese(II) mol­ecule and two chloro­benzene solvate mol­ecules. As illustrated in Fig. 1 ▸, the metal atom exhibits a five-coordinate structure (Table 1 ▸) with a significant metal out-of-plane displacement of 0.66 Å, indicative of the high-spin state of Mn^II^. Additional qu­anti­tative information on the structure is provided in supplementary Fig. 1 ▸, presenting the displacements of each porphyrin core atom from the 24-atom mean plane. Averaged values of the chemically unique bond lengths (Å) and angles (°) are also displayed. The hindered 2,5-di­methyl­imidazole ligand may also contribute to the large out-of-plane displacement for the metal atom. The dihedral angle formed by the 2,5-di­methyl­imidazole axial ligand plane and the closest Mn—N_p_ vector is 37.3°. The average N_p_—Mn—N_p_ angle is 86.0 (7)° and the axial Mn—N_Im_ bond length is 2.171 (8) Å. The average Mn—N_p_ distance of 2.143 (8) Å is a typical value for high-spin manganese porphyrin derivatives.

**Figure 2:**
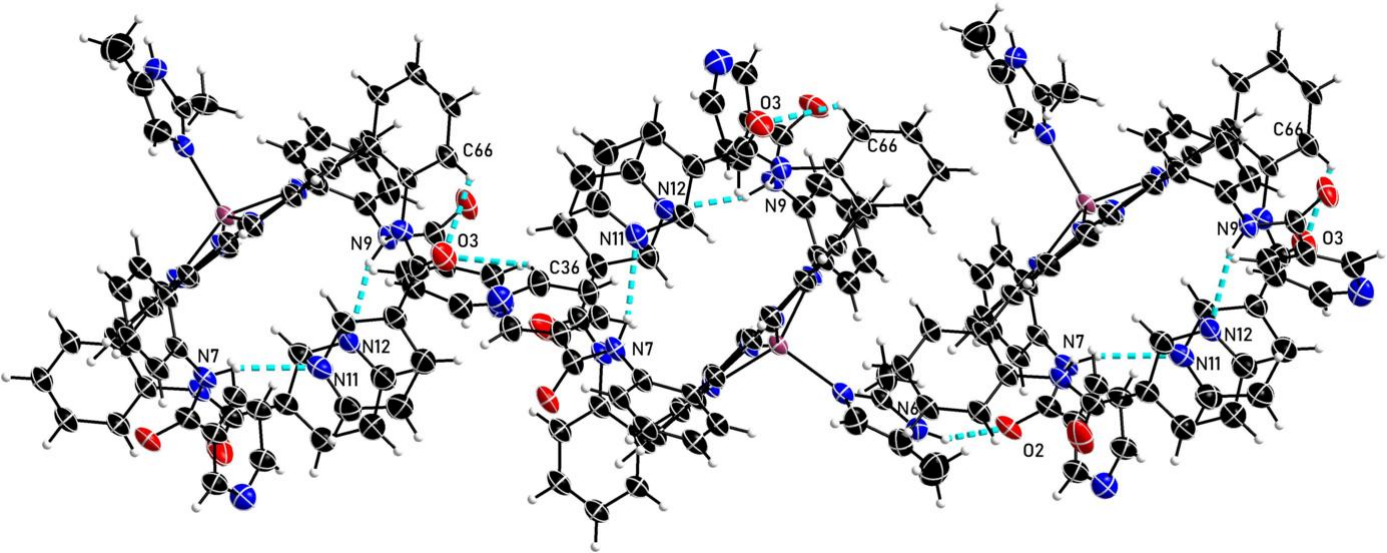
Intra- and inter-mol­ecular inter­actions in the crystal structure of the title compound.

**Figure 3:**
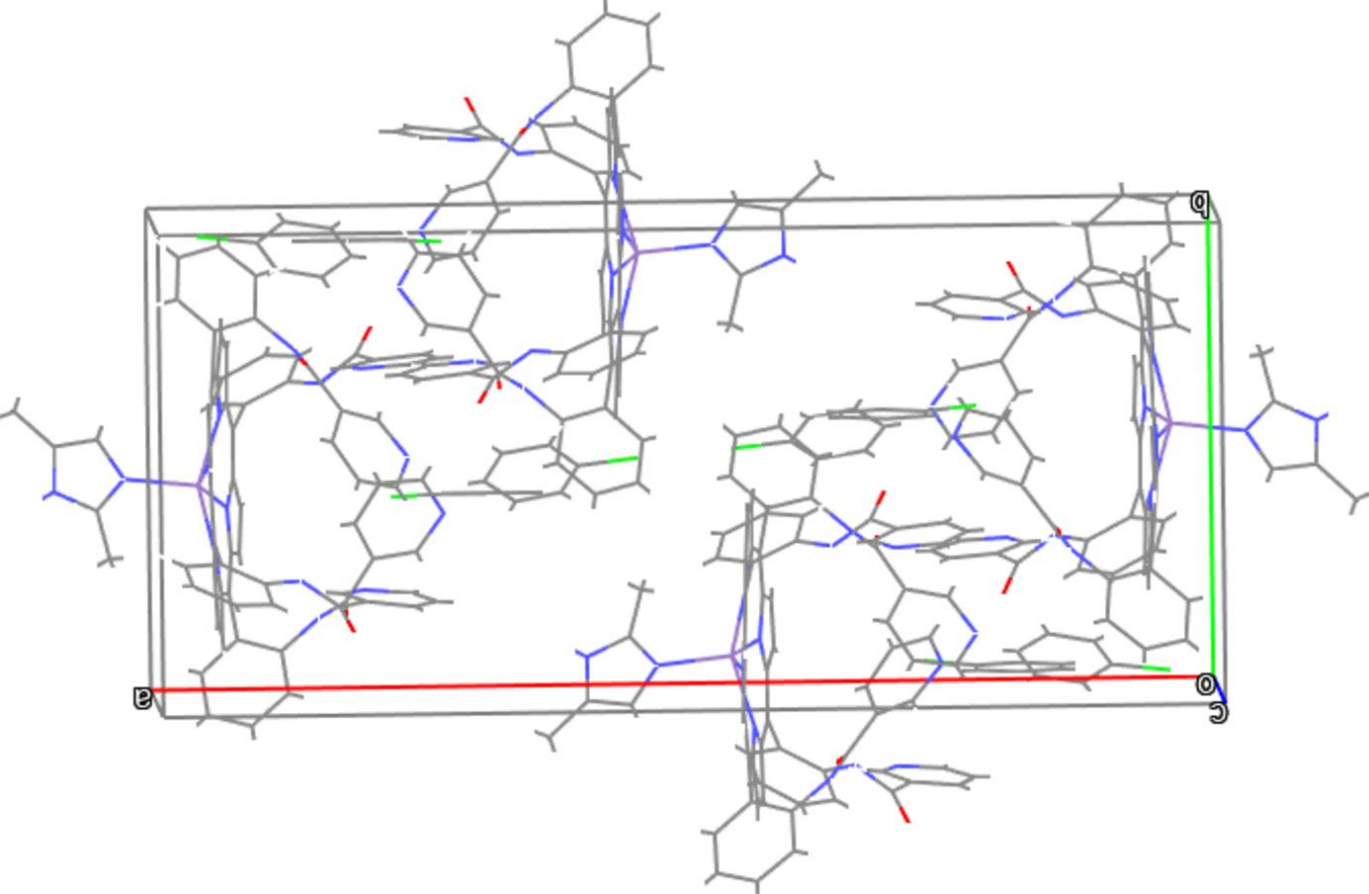
A view of the packing of the title compound. H atoms have been omitted for clarity.

Several intra- and inter-mol­ecular inter­actions are identified in the title compound (Table 2 ▸, Fig. 2 ▸): the distances between N7 and N11, N9 and N12, C42 and O4 are 3.098 (12), 3.011 (11) and 2.880 (13) Å, respectively. The distance between N6 and O2, as well as the N6—H6⋯O2 angle, are found to be 2.856 (12) Å and 153°, respectively, consistent with the N—H⋯O inter­action criteria of 2.7 < N⋯O < 3.05 Å and N—H⋯O > 130° (Landrum *et al.*, 1980 ▸). The mol­ecular packing is shown in Fig. 3 ▸.

## Synthesis and crystallization

All experimental manipulations in this work were conducted under an argon atmosphere using a double-manifold vacuum line, Schlenkware and cannula techniques. With the exception of the solvent used in column chromatography, all solvents utilized in the experimental procedures were subjected to anhydrous and anaerobic conditions. Chloro­benzene, benzene and *n*-hexane were distilled over P_2_O_5_ and potassium–sodium alloy, respectively. All solvents employed in the anhydrous and anaerobic operations (Schlenk system) underwent the freeze–pump–thaw method three times before use. The precursors H_2_(TPyPP), [Mn(TPyPP)]Cl, and [Mn(TPyPP)]OH were prepared following literature methods (Gunter *et al.*, 1984 ▸), with slight modifications.

[Mn(TPyPP)]OH (10 mg) was dried under vacuum for 30 minutes and dissolved in 5 ml of benzene. After adding 1 ml of ethane­thiol, the solution was stirred for 1 day and then evacuated under vacuum to yield a purple powder. The resulting purple solid of [Mn(TPyPP)] (10 mg) was dried for 60 minutes, and excess 2,5-di­methyl­imidazole in PhCl (5 ml) was added using a cannula. The mixture was stirred for 1 h and transferred into glass tubes, which were layered with *n*-hexane as a non-polar solvent. Several weeks later, X-ray quality crystals of the title compound in the form of black blocks were collected.

## Refinement

Crystal data, data collection and structure refinement details are summarized in Table 3 ▸. The crystal studied was refined as a 2-component inversion twin.

## Supplementary Material

Crystal structure: contains datablock(s) I. DOI: 10.1107/S2414314624004978/hb4469sup1.cif

Structure factors: contains datablock(s) I. DOI: 10.1107/S2414314624004978/hb4469Isup2.hkl

Porphyrin ring displacement data. DOI: 10.1107/S2414314624004978/hb4469sup3.docx

CCDC reference: 2358337

Additional supporting information:  crystallographic information; 3D view; checkCIF report

## Figures and Tables

**Table 1:** Selected geometric parameters (Å, °)

Mn1—N1	2.138 (6)	Mn1—N4	2.154 (6)
Mn1—N2	2.141 (6)	Mn1—N5	2.171 (8)
Mn1—N3	2.141 (6)		
			
N1—Mn1—N2	86.9 (2)	N2—Mn1—N5	101.4 (3)
N1—Mn1—N3	150.0 (3)	N3—Mn1—N2	85.3 (2)
N1—Mn1—N4	85.5 (2)	N3—Mn1—N4	86.4 (2)
N1—Mn1—N5	98.1 (3)	N3—Mn1—N5	111.8 (3)
N2—Mn1—N4	148.9 (3)	N4—Mn1—N5	109.5 (3)

**Table 2:** Hydrogen-bond geometry (Å, °)

*D*—H⋯*A*	*D*—H	H⋯*A*	*D*⋯*A*	*D*—H⋯*A*
N6—H6⋯O2^i^	0.88	2.05	2.858 (11)	153
N7—H7*A*⋯N11	0.88	2.24	3.094 (11)	164
N9—H9⋯N12	0.88	2.17	3.009 (10)	159
C5*S*—H5*S*⋯N14^ii^	0.95	2.57	3.421 (16)	150
C36—H36⋯O3^ii^	0.95	2.35	2.99 (2)	124
C60—H60⋯O4^iii^	0.95	2.40	3.062 (18)	126

Symmetry codes: (i) 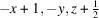; (ii) 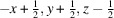; (iii) 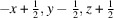.

**Table 3:** Experimental details

Crystal data	
Chemical formula	[Mn(C_68_H_44_N_12_O_4_)(C_5_H_8_N_2_)]·2C_6_H_5_Cl
*M*_r_	1469.32
Crystal system, space group	Orthorhombic, *Pna*2_1_
Temperature (K)	101
*a*, *b*, *c* (Å)	30.247 (4), 13.713 (2), 17.205 (2)
*V* (Å^3^)	7136.2 (16)
*Z*	4
Radiation type	Mo *K*α
μ (mm^−1^)	0.33
Crystal size (mm)	0.61 × 0.55 × 0.35

Data collection	
Diffractometer	Bruker APEXII CCD
Absorption correction	Multi-scan (*SADABS*; Krause *et al.*, 2015 ▸)
*T*_min_, *T*_max_	0.568, 0.745
No. of measured, independent and observed [*I* > 2σ(*I*)] reflections	142482, 14653, 12171
*R*_int_	0.071
(sin θ/λ)_max_ (Å^−1^)	0.629

Refinement	
*R*[*F*^2^ > 2σ(*F*^2^)], *wR*(*F*^2^), *S*	0.080, 0.240, 1.06
No. of reflections	14653
No. of parameters	946
No. of restraints	1
H-atom treatment	H-atom parameters constrained
Δρ_max_, Δρ_min_ (e Å^−3^)	0.98, −1.03
Absolute structure	Refined as an inversion twin.
Absolute structure parameter	0.41 (4)

Computer programs: *APEX2* and *SAINT* (Bruker, 2014 ▸), *SHELXT2018/2* (Sheldrick, 2015*a* ▸), *SHELXL2018/3* (Sheldrick, 2015*b* ▸) and *OLEX2* (Dolomanov *et al.*, 2009 ▸).

## full crystallographic data

### (2,5-Dimethylimidazole){N,N',N'',N'''-[porphyrin-5,10,15,20-tetrayltetra(2,1-phenylene)]tetrakis(pyridine-3-carboxamide)}manganese(II) chlorobenzene disolvate Crystal data

**Table:**

[Mn(C_68_H_44_N_12_O_4_)(C_5_H_8_N_2_)]·2C_6_H_5_Cl	*D*_x_ = 1.368 Mg m^−^^3^
*M**_r_* = 1469.32	Mo *K*α radiation, λ = 0.71073 Å
Orthorhombic, *P**n**a*2_1_	Cell parameters from 9672 reflections
*a* = 30.247 (4) Å	θ = 2.5–26.3°
*b* = 13.713 (2) Å	µ = 0.33 mm^−^^1^
*c* = 17.205 (2) Å	*T* = 101 K
*V* = 7136.2 (16) Å^3^	Block, black
*Z* = 4	0.61 × 0.55 × 0.35 mm
*F*(000) = 3044	

### (2,5-Dimethylimidazole){N,N',N'',N'''-[porphyrin-5,10,15,20-tetrayltetra(2,1-phenylene)]tetrakis(pyridine-3-carboxamide)}manganese(II) chlorobenzene disolvate Data collection

**Table:**

Bruker APEXII CCD diffractometer	12171 reflections with *I* > 2σ(*I*)
φ and ω scans	*R*_int_ = 0.071
Absorption correction: multi-scan (SADABS; Krause *et al.*, 2015)	θ_max_ = 26.6°, θ_min_ = 2.0°
*T*_min_ = 0.568, *T*_max_ = 0.745	*h* = −37→37
142482 measured reflections	*k* = −17→17
14653 independent reflections	*l* = −21→21

### (2,5-Dimethylimidazole){N,N',N'',N'''-[porphyrin-5,10,15,20-tetrayltetra(2,1-phenylene)]tetrakis(pyridine-3-carboxamide)}manganese(II) chlorobenzene disolvate Refinement

**Table:**

Refinement on *F*^2^	Hydrogen site location: inferred from neighbouring sites
Least-squares matrix: full	H-atom parameters constrained
*R*[*F*^2^ > 2σ(*F*^2^)] = 0.080	*w* = 1/[σ^2^(*F*_o_^2^) + (0.1079*P*)^2^ + 24.1645*P*] where *P* = (*F*_o_^2^ + 2*F*_c_^2^)/3
*wR*(*F*^2^) = 0.240	(Δ/σ)_max_ < 0.001
*S* = 1.06	Δρ_max_ = 0.98 e Å^−^^3^
14653 reflections	Δρ_min_ = −1.03 e Å^−^^3^
946 parameters	Absolute structure: Refined as an inversion twin.
1 restraint	Absolute structure parameter: 0.41 (4)
Primary atom site location: dual	

### (2,5-Dimethylimidazole){N,N',N'',N'''-[porphyrin-5,10,15,20-tetrayltetra(2,1-phenylene)]tetrakis(pyridine-3-carboxamide)}manganese(II) chlorobenzene disolvate Special details

**Table:**

Geometry. All esds (except the esd in the dihedral angle between two l.s. planes) are estimated using the full covariance matrix. The cell esds are taken into account individually in the estimation of esds in distances, angles and torsion angles; correlations between esds in cell parameters are only used when they are defined by crystal symmetry. An approximate (isotropic) treatment of cell esds is used for estimating esds involving l.s. planes.
Refinement. Refined as a 2-component inversion twin

### (2,5-Dimethylimidazole){N,N',N'',N'''-[porphyrin-5,10,15,20-tetrayltetra(2,1-phenylene)]tetrakis(pyridine-3-carboxamide)}manganese(II) chlorobenzene disolvate Fractional atomic coordinates and isotropic or equivalent isotropic displacement parameters (Å^2^)

**Table:**

	*x*	*y*	*z*	*U*_iso_*/*U*_eq_	
Mn1	0.45525 (4)	0.06614 (8)	0.17877 (7)	0.0281 (3)	
N1	0.4348 (2)	−0.0793 (4)	0.2054 (4)	0.0262 (13)	
N2	0.4298 (2)	0.1066 (5)	0.2903 (4)	0.0279 (13)	
N3	0.4393 (2)	0.2123 (5)	0.1452 (4)	0.0289 (13)	
N4	0.4429 (2)	0.0271 (5)	0.0595 (4)	0.0271 (13)	
C4	0.4360 (3)	−0.1573 (5)	0.1553 (4)	0.0251 (15)	
C1	0.4293 (3)	−0.1147 (5)	0.2795 (4)	0.0270 (15)	
C19	0.4243 (2)	0.0426 (5)	0.3515 (4)	0.0255 (14)	
C16	0.4268 (3)	0.1998 (6)	0.3193 (5)	0.0300 (16)	
C14	0.4347 (3)	0.2913 (5)	0.1942 (4)	0.0299 (16)	
C11	0.4395 (3)	0.2489 (6)	0.0699 (5)	0.0287 (15)	
C9	0.4409 (3)	0.0900 (6)	−0.0030 (4)	0.0279 (15)	
C6	0.4424 (3)	−0.0655 (6)	0.0300 (4)	0.0292 (16)	
C3	0.4323 (3)	−0.2477 (5)	0.2004 (4)	0.0295 (16)	
H3	0.432566	−0.312405	0.180631	0.035*	
C2	0.4283 (3)	−0.2206 (6)	0.2763 (5)	0.0318 (17)	
H2	0.425412	−0.263366	0.319432	0.038*	
C18	0.4181 (3)	0.0991 (6)	0.4221 (4)	0.0339 (18)	
H18	0.413455	0.073443	0.472722	0.041*	
C17	0.4200 (3)	0.1945 (6)	0.4031 (4)	0.0321 (17)	
H17	0.417493	0.248084	0.437833	0.038*	
C13	0.4340 (3)	0.3799 (6)	0.1479 (5)	0.0344 (17)	
H13	0.431639	0.444585	0.167184	0.041*	
C12	0.4372 (3)	0.3535 (6)	0.0724 (5)	0.0335 (17)	
H12	0.437852	0.396232	0.028974	0.040*	
C8	0.4404 (3)	0.0337 (6)	−0.0748 (5)	0.0343 (18)	
H8	0.439129	0.059094	−0.126106	0.041*	
C7	0.4419 (3)	−0.0621 (6)	−0.0543 (5)	0.0337 (17)	
H7	0.442578	−0.116402	−0.088618	0.040*	
C5	0.4391 (3)	−0.1523 (6)	0.0742 (4)	0.0286 (16)	
C20	0.4238 (3)	−0.0582 (6)	0.3470 (4)	0.0279 (15)	
C15	0.4287 (3)	0.2852 (6)	0.2751 (4)	0.0287 (16)	
C10	0.4390 (3)	0.1915 (5)	0.0011 (5)	0.0285 (15)	
C38	0.4317 (3)	0.2450 (6)	−0.0742 (4)	0.0328 (17)	
C43	0.3902 (3)	0.2874 (6)	−0.0896 (5)	0.0346 (17)	
C42	0.3823 (3)	0.3348 (7)	−0.1599 (5)	0.041 (2)	
H42	0.354400	0.363857	−0.170375	0.049*	
C41	0.4162 (4)	0.3385 (7)	−0.2143 (5)	0.043 (2)	
H41	0.411037	0.370699	−0.262340	0.052*	
C40	0.4568 (3)	0.2973 (7)	−0.2011 (5)	0.041 (2)	
H40	0.479286	0.299728	−0.239652	0.050*	
C39	0.4645 (3)	0.2518 (6)	−0.1300 (5)	0.0370 (18)	
H39	0.492859	0.224862	−0.119639	0.044*	
C26	0.4362 (3)	−0.2455 (6)	0.0290 (4)	0.0299 (16)	
C31	0.3979 (3)	−0.2690 (6)	−0.0133 (5)	0.0333 (17)	
C30	0.3956 (3)	−0.3541 (6)	−0.0573 (5)	0.0387 (19)	
H30	0.369625	−0.368807	−0.085973	0.046*	
C29	0.4311 (3)	−0.4166 (6)	−0.0592 (5)	0.041 (2)	
H29	0.429615	−0.474211	−0.089785	0.049*	
C28	0.4693 (4)	−0.3962 (6)	−0.0166 (5)	0.043 (2)	
H28	0.493690	−0.439754	−0.018096	0.052*	
C27	0.4714 (3)	−0.3119 (6)	0.0279 (5)	0.0342 (17)	
H27	0.497053	−0.299127	0.058142	0.041*	
C62	0.4133 (3)	−0.1143 (5)	0.4194 (4)	0.0289 (16)	
C67	0.3725 (3)	−0.1613 (6)	0.4269 (4)	0.0318 (17)	
C66	0.3630 (3)	−0.2179 (7)	0.4948 (5)	0.041 (2)	
H66	0.335455	−0.250347	0.500374	0.049*	
C65	0.3950 (3)	−0.2241 (6)	0.5522 (5)	0.040 (2)	
H65	0.389080	−0.261811	0.597376	0.048*	
C64	0.4355 (3)	−0.1768 (7)	0.5457 (5)	0.039 (2)	
H64	0.457141	−0.182661	0.585507	0.047*	
C63	0.4439 (3)	−0.1204 (6)	0.4797 (5)	0.0334 (17)	
H63	0.470918	−0.085468	0.475856	0.040*	
C50	0.4216 (3)	0.3793 (5)	0.3174 (5)	0.0304 (16)	
C55	0.3806 (3)	0.4020 (6)	0.3517 (5)	0.0332 (17)	
C54	0.3749 (3)	0.4881 (6)	0.3924 (5)	0.0395 (19)	
H54	0.347039	0.502786	0.414984	0.047*	
C53	0.4101 (4)	0.5537 (6)	0.4006 (5)	0.043 (2)	
H53	0.406239	0.612243	0.429356	0.052*	
C52	0.4507 (3)	0.5327 (6)	0.3662 (5)	0.041 (2)	
H52	0.474763	0.576813	0.371583	0.049*	
C51	0.4560 (3)	0.4474 (6)	0.3244 (5)	0.0343 (18)	
H51	0.483525	0.434614	0.299801	0.041*	
C21	0.5500 (3)	−0.0324 (7)	0.1896 (7)	0.050 (2)	
H21	0.539801	−0.088773	0.163089	0.059*	
C22	0.5911 (4)	−0.0208 (8)	0.2188 (6)	0.055 (3)	
C23	0.6282 (5)	−0.0939 (14)	0.2290 (11)	0.099 (5)	
H23A	0.654723	−0.070274	0.202121	0.148*	
H23B	0.619323	−0.156791	0.206977	0.148*	
H23C	0.634676	−0.101800	0.284452	0.148*	
C24	0.5516 (3)	0.1122 (6)	0.2425 (5)	0.0347 (17)	
C25	0.5403 (3)	0.2105 (7)	0.2705 (6)	0.044 (2)	
H25A	0.565198	0.236989	0.300271	0.066*	
H25B	0.514134	0.206798	0.303958	0.066*	
H25C	0.534110	0.253002	0.226096	0.066*	
C44	0.3205 (3)	0.3335 (7)	−0.0220 (5)	0.042 (2)	
C45	0.2967 (3)	0.3183 (7)	0.0538 (5)	0.0381 (19)	
C49	0.3200 (3)	0.2978 (7)	0.1214 (5)	0.042 (2)	
H49	0.351180	0.291514	0.118252	0.051*	
C46	0.2518 (4)	0.3300 (9)	0.0590 (6)	0.054 (3)	
H46	0.235080	0.347755	0.014461	0.064*	
C47	0.2312 (4)	0.3157 (12)	0.1291 (7)	0.069 (4)	
H47	0.199932	0.321103	0.133299	0.082*	
C48	0.2562 (4)	0.2938 (10)	0.1924 (6)	0.060 (3)	
H48	0.241461	0.283059	0.240442	0.072*	
C32	0.3453 (3)	−0.1570 (7)	−0.0738 (5)	0.040 (2)	
C33	0.3165 (4)	−0.0722 (7)	−0.0565 (6)	0.046 (2)	
C34	0.3268 (4)	−0.0108 (7)	0.0064 (6)	0.055 (3)	
H34	0.351483	−0.025289	0.038401	0.065*	
C37	0.2820 (5)	−0.0483 (8)	−0.1046 (7)	0.068 (4)	
H37	0.276962	−0.085239	−0.150433	0.081*	
C35	0.3018 (5)	0.0688 (8)	0.0218 (7)	0.077 (5)	
H35	0.308722	0.110647	0.064047	0.093*	
C36	0.2662 (8)	0.0871 (11)	−0.0256 (9)	0.117 (8)	
H36	0.248724	0.143239	−0.015163	0.141*	
C68	0.3074 (3)	−0.2106 (7)	0.3468 (5)	0.042 (2)	
C69	0.2886 (3)	−0.1948 (7)	0.2677 (5)	0.0401 (19)	
C73	0.3156 (3)	−0.1769 (6)	0.2041 (5)	0.0387 (19)	
H73	0.346403	−0.169205	0.212802	0.046*	
C70	0.2435 (4)	−0.2042 (11)	0.2552 (7)	0.066 (3)	
H70	0.223972	−0.218568	0.296836	0.079*	
C71	0.2279 (4)	−0.1921 (12)	0.1808 (8)	0.078 (4)	
H71	0.196986	−0.194992	0.171200	0.094*	
C72	0.2571 (4)	−0.1758 (9)	0.1193 (7)	0.059 (3)	
H72	0.245625	−0.168762	0.068217	0.071*	
C56	0.3202 (3)	0.2986 (7)	0.4006 (5)	0.0395 (19)	
C57	0.2937 (3)	0.2114 (6)	0.3789 (5)	0.0370 (18)	
C58	0.3101 (4)	0.1405 (7)	0.3272 (6)	0.050 (2)	
H58	0.337908	0.149512	0.302639	0.060*	
C61	0.2534 (4)	0.1957 (8)	0.4125 (7)	0.058 (3)	
H61	0.243320	0.241724	0.449769	0.070*	
C59	0.2858 (6)	0.0588 (9)	0.3128 (7)	0.080 (5)	
H59	0.296744	0.009203	0.279320	0.096*	
C60	0.2464 (6)	0.0501 (10)	0.3467 (8)	0.094 (6)	
H60	0.230164	−0.007726	0.336369	0.113*	
C1S	0.3496 (2)	0.5649 (7)	0.1213 (5)	0.065 (3)	
H1S	0.377136	0.566780	0.094747	0.078*	
C5S	0.3103 (3)	0.5676 (7)	0.0795 (5)	0.101 (6)	
H5S	0.310965	0.571380	0.024379	0.121*	
C4S	0.2700 (2)	0.5649 (7)	0.1184 (7)	0.122 (9)	
C2S	0.2691 (3)	0.5594 (8)	0.1990 (7)	0.088 (5)	
H2S	0.241542	0.557478	0.225562	0.106*	
C6S	0.3084 (3)	0.5566 (8)	0.2408 (5)	0.100 (6)	
H6S	0.307712	0.552877	0.295932	0.120*	
C3S	0.3486 (3)	0.5594 (7)	0.2020 (5)	0.084 (5)	
H3S	0.375509	0.557528	0.230526	0.101*	
C7S	0.0972 (4)	0.0367 (8)	0.1522 (6)	0.052 (2)	
C8S	0.1144 (4)	0.0883 (8)	0.2146 (6)	0.058 (3)	
H8S	0.095431	0.111217	0.254540	0.069*	
C9S	0.1574 (5)	0.1054 (10)	0.2181 (8)	0.073 (4)	
H9S	0.168861	0.144867	0.258808	0.088*	
C10S	0.1236 (4)	−0.0035 (9)	0.0967 (6)	0.057 (3)	
H10S	0.111343	−0.041601	0.055922	0.068*	
C11S	0.1681 (5)	0.0125 (12)	0.1011 (8)	0.076 (4)	
H11S	0.187073	−0.012897	0.062139	0.091*	
C12S	0.1858 (5)	0.0665 (14)	0.1633 (9)	0.093 (5)	
H12S	0.216837	0.076154	0.167499	0.111*	
Cl1S	0.22099 (16)	0.5730 (4)	0.0715 (3)	0.1114 (16)	
Cl2S	0.04023 (12)	0.0223 (4)	0.1439 (2)	0.0998 (15)	
N5	0.5252 (2)	0.0496 (5)	0.2041 (4)	0.0356 (15)	
N6	0.5922 (3)	0.0716 (6)	0.2505 (5)	0.0437 (18)	
H6	0.615329	0.099384	0.272268	0.052*	
N7	0.3617 (2)	−0.2033 (5)	−0.0110 (4)	0.0355 (15)	
H7A	0.349239	−0.192075	0.034294	0.043*	
N8	0.3568 (3)	0.2791 (5)	−0.0313 (4)	0.0365 (15)	
H8A	0.360605	0.232130	0.002861	0.044*	
N9	0.3452 (2)	0.3352 (5)	0.3425 (4)	0.0347 (15)	
H9	0.339016	0.315991	0.294934	0.042*	
N10	0.3418 (2)	−0.1535 (5)	0.3659 (4)	0.0355 (15)	
H10	0.345720	−0.102892	0.335286	0.043*	
N11	0.3005 (3)	−0.1697 (6)	0.1306 (5)	0.0476 (19)	
N12	0.3006 (3)	0.2865 (7)	0.1911 (4)	0.0485 (19)	
N13	0.2545 (6)	0.0296 (9)	−0.0866 (7)	0.101 (5)	
N14	0.2268 (4)	0.1181 (9)	0.3960 (8)	0.085 (4)	
O1	0.3205 (3)	0.3298 (6)	0.4678 (4)	0.0564 (19)	
O2	0.3549 (2)	−0.1787 (5)	−0.1415 (4)	0.0496 (17)	
O3	0.2929 (4)	−0.2738 (8)	0.3903 (5)	0.091 (4)	
O4	0.3066 (3)	0.3919 (8)	−0.0691 (5)	0.082 (3)	

### (2,5-Dimethylimidazole){N,N',N'',N'''-[porphyrin-5,10,15,20-tetrayltetra(2,1-phenylene)]tetrakis(pyridine-3-carboxamide)}manganese(II) chlorobenzene disolvate Atomic displacement parameters (Å^2^)

**Table:**

	*U*^11^	*U*^22^	*U*^33^	*U*^12^	*U*^13^	*U*^23^
Mn1	0.0395 (6)	0.0223 (5)	0.0227 (5)	−0.0016 (4)	0.0000 (5)	0.0008 (5)
N1	0.041 (4)	0.017 (3)	0.021 (3)	−0.003 (2)	−0.002 (2)	−0.002 (2)
N2	0.043 (4)	0.021 (3)	0.020 (3)	−0.003 (3)	0.003 (3)	0.001 (2)
N3	0.044 (4)	0.022 (3)	0.021 (3)	0.001 (3)	−0.003 (3)	0.001 (3)
N4	0.039 (3)	0.022 (3)	0.020 (3)	0.000 (3)	−0.001 (3)	0.001 (2)
C4	0.041 (4)	0.011 (3)	0.023 (3)	0.000 (3)	−0.001 (3)	0.000 (3)
C1	0.035 (4)	0.021 (4)	0.025 (4)	−0.004 (3)	0.001 (3)	0.002 (3)
C19	0.034 (4)	0.022 (3)	0.020 (3)	0.001 (3)	−0.001 (3)	−0.002 (3)
C16	0.045 (4)	0.022 (4)	0.023 (4)	0.004 (3)	0.001 (3)	0.006 (3)
C14	0.042 (4)	0.023 (3)	0.025 (4)	−0.001 (3)	−0.002 (3)	−0.002 (3)
C11	0.039 (4)	0.022 (4)	0.026 (4)	0.000 (3)	−0.001 (3)	0.005 (3)
C9	0.034 (4)	0.028 (4)	0.021 (3)	0.000 (3)	0.001 (3)	0.002 (3)
C6	0.041 (4)	0.028 (4)	0.019 (3)	−0.003 (3)	−0.003 (3)	−0.003 (3)
C3	0.048 (4)	0.016 (3)	0.025 (4)	−0.005 (3)	0.002 (3)	0.004 (3)
C2	0.045 (4)	0.024 (4)	0.026 (4)	−0.002 (3)	0.002 (3)	0.004 (3)
C18	0.057 (5)	0.026 (4)	0.019 (4)	0.000 (3)	0.003 (3)	0.001 (3)
C17	0.055 (5)	0.021 (4)	0.019 (4)	0.003 (3)	−0.002 (3)	−0.005 (3)
C13	0.044 (4)	0.028 (4)	0.031 (4)	−0.005 (3)	−0.004 (3)	0.000 (3)
C12	0.049 (5)	0.018 (4)	0.034 (4)	−0.003 (3)	−0.003 (4)	0.002 (3)
C8	0.050 (5)	0.031 (4)	0.022 (4)	0.003 (4)	0.000 (3)	0.004 (3)
C7	0.048 (5)	0.030 (4)	0.024 (4)	−0.001 (3)	0.001 (3)	−0.004 (3)
C5	0.036 (4)	0.027 (4)	0.023 (4)	−0.004 (3)	−0.001 (3)	0.000 (3)
C20	0.034 (4)	0.031 (4)	0.019 (3)	−0.002 (3)	−0.001 (3)	0.002 (3)
C15	0.039 (4)	0.023 (4)	0.024 (4)	0.004 (3)	−0.003 (3)	0.001 (3)
C10	0.038 (4)	0.024 (4)	0.024 (4)	−0.001 (3)	−0.002 (3)	0.008 (3)
C38	0.053 (5)	0.024 (4)	0.021 (4)	−0.003 (3)	0.000 (3)	0.006 (3)
C43	0.048 (5)	0.027 (4)	0.028 (4)	−0.001 (3)	0.001 (4)	0.003 (3)
C42	0.053 (5)	0.037 (5)	0.032 (4)	0.002 (4)	−0.002 (4)	0.012 (4)
C41	0.068 (6)	0.037 (5)	0.025 (4)	−0.003 (4)	0.000 (4)	0.004 (3)
C40	0.056 (5)	0.036 (4)	0.032 (4)	−0.001 (4)	0.006 (4)	0.006 (4)
C39	0.045 (4)	0.029 (4)	0.038 (4)	0.000 (3)	0.005 (4)	0.004 (3)
C26	0.042 (4)	0.028 (4)	0.020 (3)	−0.002 (3)	0.002 (3)	−0.003 (3)
C31	0.050 (5)	0.025 (4)	0.025 (4)	−0.003 (3)	−0.005 (3)	−0.002 (3)
C30	0.059 (5)	0.030 (4)	0.027 (4)	−0.003 (4)	−0.004 (4)	−0.009 (3)
C29	0.065 (6)	0.027 (4)	0.032 (4)	−0.001 (4)	0.002 (4)	−0.005 (3)
C28	0.070 (6)	0.026 (4)	0.033 (4)	0.005 (4)	0.005 (4)	−0.003 (3)
C27	0.047 (5)	0.026 (4)	0.030 (4)	−0.001 (3)	−0.001 (3)	0.000 (3)
C62	0.044 (4)	0.024 (4)	0.019 (3)	0.002 (3)	0.000 (3)	0.001 (3)
C67	0.042 (4)	0.031 (4)	0.022 (4)	−0.001 (3)	0.003 (3)	0.008 (3)
C66	0.053 (5)	0.037 (4)	0.032 (4)	−0.003 (4)	0.006 (4)	0.011 (4)
C65	0.068 (6)	0.032 (4)	0.021 (4)	−0.001 (4)	0.003 (4)	0.004 (3)
C64	0.061 (6)	0.036 (4)	0.021 (4)	0.008 (4)	0.000 (4)	0.003 (3)
C63	0.042 (4)	0.034 (4)	0.024 (4)	−0.004 (3)	−0.003 (3)	0.001 (3)
C50	0.048 (5)	0.015 (3)	0.028 (4)	0.000 (3)	−0.001 (3)	0.000 (3)
C55	0.046 (4)	0.021 (3)	0.032 (4)	−0.003 (3)	0.001 (3)	0.000 (3)
C54	0.059 (5)	0.022 (4)	0.037 (5)	0.004 (4)	0.010 (4)	−0.003 (3)
C53	0.070 (6)	0.021 (4)	0.038 (5)	−0.005 (4)	0.008 (4)	−0.008 (3)
C52	0.061 (6)	0.025 (4)	0.036 (5)	−0.002 (4)	−0.002 (4)	0.000 (3)
C51	0.050 (5)	0.019 (4)	0.034 (4)	−0.001 (3)	0.001 (3)	0.000 (3)
C21	0.060 (6)	0.039 (4)	0.050 (6)	0.017 (4)	−0.009 (5)	−0.005 (4)
C22	0.065 (7)	0.055 (6)	0.043 (6)	0.018 (5)	−0.007 (5)	−0.015 (5)
C23	0.064 (9)	0.117 (14)	0.115 (13)	0.024 (9)	−0.013 (9)	−0.025 (11)
C24	0.046 (5)	0.033 (4)	0.025 (4)	0.000 (3)	0.001 (3)	0.006 (3)
C25	0.054 (5)	0.033 (4)	0.044 (5)	−0.004 (4)	−0.015 (4)	0.007 (4)
C44	0.048 (5)	0.046 (5)	0.032 (5)	0.006 (4)	−0.003 (4)	0.008 (4)
C45	0.045 (5)	0.039 (5)	0.031 (4)	0.000 (4)	−0.003 (4)	0.003 (4)
C49	0.050 (5)	0.042 (5)	0.034 (4)	−0.004 (4)	0.000 (4)	0.005 (4)
C46	0.053 (6)	0.074 (7)	0.034 (5)	0.004 (5)	−0.002 (4)	−0.005 (5)
C47	0.041 (5)	0.117 (11)	0.048 (6)	−0.003 (6)	0.001 (5)	−0.012 (7)
C48	0.057 (6)	0.087 (8)	0.036 (6)	−0.017 (6)	−0.001 (4)	−0.006 (5)
C32	0.058 (5)	0.033 (4)	0.030 (4)	0.003 (4)	−0.007 (4)	−0.001 (3)
C33	0.067 (6)	0.030 (4)	0.039 (5)	0.005 (4)	−0.017 (5)	0.004 (4)
C34	0.085 (8)	0.031 (5)	0.048 (6)	0.015 (5)	−0.029 (5)	−0.008 (4)
C37	0.104 (10)	0.043 (6)	0.056 (7)	0.020 (6)	−0.040 (7)	−0.007 (5)
C35	0.141 (13)	0.031 (5)	0.060 (7)	0.032 (6)	−0.055 (8)	−0.017 (5)
C36	0.21 (2)	0.067 (9)	0.075 (10)	0.075 (12)	−0.072 (12)	−0.023 (8)
C68	0.047 (5)	0.042 (5)	0.036 (5)	−0.015 (4)	0.005 (4)	0.005 (4)
C69	0.048 (5)	0.038 (5)	0.035 (5)	−0.002 (4)	−0.001 (4)	−0.010 (4)
C73	0.051 (5)	0.033 (4)	0.032 (4)	0.001 (4)	−0.003 (4)	0.005 (3)
C70	0.047 (6)	0.106 (10)	0.045 (6)	−0.013 (6)	0.002 (5)	−0.022 (6)
C71	0.041 (5)	0.132 (12)	0.062 (7)	0.006 (6)	−0.008 (6)	−0.038 (9)
C72	0.060 (6)	0.068 (7)	0.051 (6)	0.017 (6)	−0.008 (5)	−0.024 (5)
C56	0.046 (5)	0.043 (5)	0.030 (4)	0.002 (4)	0.004 (4)	−0.003 (4)
C57	0.058 (5)	0.024 (4)	0.029 (4)	−0.001 (3)	0.005 (4)	0.005 (3)
C58	0.076 (7)	0.032 (5)	0.041 (5)	−0.010 (5)	0.010 (5)	−0.005 (4)
C61	0.068 (7)	0.047 (6)	0.059 (7)	−0.016 (5)	0.018 (6)	−0.013 (5)
C59	0.144 (13)	0.040 (6)	0.056 (7)	−0.037 (7)	0.040 (8)	−0.020 (5)
C60	0.171 (17)	0.051 (7)	0.060 (8)	−0.060 (9)	0.029 (9)	−0.008 (6)
C1S	0.057 (7)	0.060 (7)	0.077 (8)	0.003 (5)	−0.007 (6)	0.019 (6)
C5S	0.094 (12)	0.062 (9)	0.146 (17)	0.009 (8)	−0.003 (11)	−0.005 (10)
C4S	0.076 (10)	0.041 (7)	0.25 (3)	−0.005 (6)	0.030 (13)	0.032 (11)
C2S	0.065 (8)	0.127 (14)	0.072 (10)	−0.005 (8)	0.006 (7)	0.031 (10)
C6S	0.097 (12)	0.102 (13)	0.101 (13)	0.011 (10)	0.028 (10)	0.038 (11)
C3S	0.082 (9)	0.055 (7)	0.114 (14)	−0.009 (6)	−0.005 (8)	0.021 (8)
C7S	0.067 (6)	0.051 (6)	0.039 (5)	0.010 (5)	0.004 (5)	0.016 (4)
C8S	0.082 (8)	0.050 (6)	0.040 (6)	0.019 (6)	0.007 (5)	0.010 (5)
C9S	0.102 (11)	0.065 (8)	0.052 (7)	−0.004 (7)	−0.005 (7)	−0.019 (6)
C10S	0.076 (8)	0.058 (6)	0.037 (5)	0.004 (6)	0.001 (5)	−0.001 (5)
C11S	0.077 (9)	0.103 (11)	0.048 (7)	0.019 (8)	0.004 (6)	−0.012 (7)
C12S	0.075 (9)	0.128 (14)	0.075 (10)	−0.009 (9)	0.003 (7)	−0.026 (9)
Cl1S	0.085 (3)	0.131 (4)	0.118 (4)	−0.001 (3)	−0.002 (3)	0.021 (3)
Cl2S	0.0616 (18)	0.170 (5)	0.068 (2)	0.012 (2)	−0.0020 (16)	0.030 (3)
N5	0.045 (4)	0.034 (4)	0.029 (3)	−0.002 (3)	0.001 (3)	0.001 (3)
N6	0.039 (4)	0.055 (5)	0.038 (4)	0.003 (3)	−0.004 (3)	−0.003 (4)
N7	0.051 (4)	0.031 (3)	0.024 (3)	0.002 (3)	−0.004 (3)	0.001 (3)
N8	0.049 (4)	0.035 (4)	0.026 (3)	0.001 (3)	−0.001 (3)	0.007 (3)
N9	0.047 (4)	0.030 (3)	0.027 (3)	−0.004 (3)	0.005 (3)	−0.003 (3)
N10	0.043 (4)	0.030 (4)	0.034 (4)	−0.010 (3)	−0.003 (3)	0.004 (3)
N11	0.056 (5)	0.051 (5)	0.036 (4)	0.008 (4)	−0.006 (4)	0.001 (4)
N12	0.055 (5)	0.063 (5)	0.028 (4)	−0.011 (4)	0.003 (3)	0.002 (4)
N13	0.163 (13)	0.070 (8)	0.071 (7)	0.059 (8)	−0.047 (8)	−0.023 (6)
N14	0.109 (9)	0.066 (7)	0.080 (8)	−0.043 (7)	0.026 (7)	−0.012 (6)
O1	0.077 (5)	0.059 (4)	0.033 (4)	−0.019 (4)	0.016 (3)	−0.015 (3)
O2	0.069 (5)	0.056 (4)	0.023 (3)	0.002 (3)	−0.007 (3)	−0.002 (3)
O3	0.136 (9)	0.099 (7)	0.038 (4)	−0.084 (7)	−0.013 (5)	0.015 (4)
O4	0.098 (7)	0.102 (7)	0.045 (4)	0.056 (6)	0.017 (4)	0.031 (5)

### (2,5-Dimethylimidazole){N,N',N'',N'''-[porphyrin-5,10,15,20-tetrayltetra(2,1-phenylene)]tetrakis(pyridine-3-carboxamide)}manganese(II) chlorobenzene disolvate Geometric parameters (Å, º)

**Table:**

Mn1—N1	2.138 (6)	C22—C23	1.515 (17)
Mn1—N2	2.141 (6)	C22—N6	1.380 (13)
Mn1—N3	2.141 (6)	C23—H23A	0.9800
Mn1—N4	2.154 (6)	C23—H23B	0.9800
Mn1—N5	2.171 (8)	C23—H23C	0.9800
N1—C4	1.373 (9)	C24—C25	1.471 (13)
N1—C1	1.374 (10)	C24—N5	1.347 (11)
N2—C19	1.379 (10)	C24—N6	1.356 (12)
N2—C16	1.374 (10)	C25—H25A	0.9800
N3—C14	1.380 (10)	C25—H25B	0.9800
N3—C11	1.389 (10)	C25—H25C	0.9800
N4—C9	1.380 (10)	C44—C45	1.504 (13)
N4—C6	1.367 (10)	C44—N8	1.336 (12)
C4—C3	1.467 (9)	C44—O4	1.214 (12)
C4—C5	1.402 (10)	C45—C49	1.388 (13)
C1—C2	1.454 (11)	C45—C46	1.371 (14)
C1—C20	1.406 (11)	C49—H49	0.9500
C19—C18	1.453 (10)	C49—N12	1.344 (12)
C19—C20	1.385 (11)	C46—H46	0.9500
C16—C17	1.459 (10)	C46—C47	1.371 (16)
C16—C15	1.398 (11)	C47—H47	0.9500
C14—C13	1.452 (11)	C47—C48	1.361 (16)
C14—C15	1.406 (11)	C48—H48	0.9500
C11—C12	1.436 (11)	C48—N12	1.347 (14)
C11—C10	1.421 (11)	C32—C33	1.482 (13)
C9—C8	1.455 (11)	C32—N7	1.349 (11)
C9—C10	1.395 (11)	C32—O2	1.237 (11)
C6—C7	1.452 (11)	C33—C34	1.406 (13)
C6—C5	1.416 (11)	C33—C37	1.371 (14)
C3—H3	0.9500	C34—H34	0.9500
C3—C2	1.362 (11)	C34—C35	1.352 (15)
C2—H2	0.9500	C37—H37	0.9500
C18—H18	0.9500	C37—N13	1.391 (16)
C18—C17	1.349 (11)	C35—H35	0.9500
C17—H17	0.9500	C35—C36	1.376 (19)
C13—H13	0.9500	C36—H36	0.9500
C13—C12	1.352 (12)	C36—N13	1.360 (18)
C12—H12	0.9500	C68—C69	1.491 (13)
C8—H8	0.9500	C68—N10	1.341 (11)
C8—C7	1.362 (12)	C68—O3	1.226 (12)
C7—H7	0.9500	C69—C73	1.388 (13)
C5—C26	1.498 (11)	C69—C70	1.386 (14)
C20—C62	1.499 (10)	C73—H73	0.9500
C15—C50	1.497 (11)	C73—N11	1.349 (12)
C10—C38	1.505 (10)	C70—H70	0.9500
C38—C43	1.409 (12)	C70—C71	1.373 (18)
C38—C39	1.386 (12)	C71—H71	0.9500
C43—C42	1.394 (12)	C71—C72	1.396 (18)
C43—N8	1.427 (11)	C72—H72	0.9500
C42—H42	0.9500	C72—N11	1.329 (14)
C42—C41	1.389 (14)	C56—C57	1.488 (12)
C41—H41	0.9500	C56—N9	1.350 (11)
C41—C40	1.373 (14)	C56—O1	1.232 (11)
C40—H40	0.9500	C57—C58	1.408 (13)
C40—C39	1.392 (12)	C57—C61	1.365 (14)
C39—H39	0.9500	C58—H58	0.9500
C26—C31	1.406 (11)	C58—C59	1.362 (15)
C26—C27	1.400 (12)	C61—H61	0.9500
C31—C30	1.393 (11)	C61—N14	1.364 (15)
C31—N7	1.417 (11)	C59—H59	0.9500
C30—H30	0.9500	C59—C60	1.33 (2)
C30—C29	1.373 (13)	C60—H60	0.9500
C29—H29	0.9500	C60—N14	1.39 (2)
C29—C28	1.397 (14)	C1S—H1S	0.9500
C28—H28	0.9500	C1S—C5S	1.3900
C28—C27	1.388 (12)	C1S—C3S	1.3900
C27—H27	0.9500	C5S—H5S	0.9500
C62—C67	1.399 (11)	C5S—C4S	1.3900
C62—C63	1.391 (11)	C4S—C2S	1.3900
C67—C66	1.432 (11)	C4S—Cl1S	1.692 (8)
C67—N10	1.405 (11)	C2S—H2S	0.9500
C66—H66	0.9500	C2S—C6S	1.3900
C66—C65	1.385 (13)	C6S—H6S	0.9500
C65—H65	0.9500	C6S—C3S	1.3900
C65—C64	1.392 (14)	C3S—H3S	0.9500
C64—H64	0.9500	C7S—C8S	1.387 (16)
C64—C63	1.398 (12)	C7S—C10S	1.360 (15)
C63—H63	0.9500	C7S—Cl2S	1.740 (12)
C50—C55	1.409 (12)	C8S—H8S	0.9500
C50—C51	1.404 (12)	C8S—C9S	1.322 (19)
C55—C54	1.384 (11)	C9S—H9S	0.9500
C55—N9	1.417 (11)	C9S—C12S	1.38 (2)
C54—H54	0.9500	C10S—H10S	0.9500
C54—C53	1.399 (14)	C10S—C11S	1.366 (19)
C53—H53	0.9500	C11S—H11S	0.9500
C53—C52	1.395 (14)	C11S—C12S	1.41 (2)
C52—H52	0.9500	C12S—H12S	0.9500
C52—C51	1.382 (12)	N6—H6	0.8800
C51—H51	0.9500	N7—N11	3.098 (12)
C21—H21	0.9500	N8—H8A	0.8800
C21—C22	1.349 (15)	N9—H9	0.8800
C21—N5	1.374 (11)	N10—H10	0.8800
			
N1—Mn1—N2	86.9 (2)	C52—C51—H51	119.3
N1—Mn1—N3	150.0 (3)	C22—C21—H21	125.0
N1—Mn1—N4	85.5 (2)	C22—C21—N5	109.9 (9)
N1—Mn1—N5	98.1 (3)	N5—C21—H21	125.0
N2—Mn1—N4	148.9 (3)	C21—C22—C23	130.4 (11)
N2—Mn1—N5	101.4 (3)	C21—C22—N6	106.1 (9)
N3—Mn1—N2	85.3 (2)	N6—C22—C23	122.9 (11)
N3—Mn1—N4	86.4 (2)	C22—C23—H23A	109.5
N3—Mn1—N5	111.8 (3)	C22—C23—H23B	109.5
N4—Mn1—N5	109.5 (3)	C22—C23—H23C	109.5
C4—N1—Mn1	125.8 (5)	H23A—C23—H23B	109.5
C4—N1—C1	108.0 (6)	H23A—C23—H23C	109.5
C1—N1—Mn1	124.3 (5)	H23B—C23—H23C	109.5
C19—N2—Mn1	124.2 (5)	N5—C24—C25	127.4 (8)
C16—N2—Mn1	126.1 (5)	N5—C24—N6	109.0 (8)
C16—N2—C19	107.9 (6)	N6—C24—C25	123.6 (8)
C14—N3—Mn1	126.3 (5)	C24—C25—H25A	109.5
C14—N3—C11	106.7 (6)	C24—C25—H25B	109.5
C11—N3—Mn1	126.1 (5)	C24—C25—H25C	109.5
C9—N4—Mn1	126.5 (5)	H25A—C25—H25B	109.5
C6—N4—Mn1	125.9 (5)	H25A—C25—H25C	109.5
C6—N4—C9	106.9 (6)	H25B—C25—H25C	109.5
N1—C4—C3	109.0 (6)	N8—C44—C45	114.9 (8)
N1—C4—C5	126.0 (7)	O4—C44—C45	120.3 (9)
C5—C4—C3	125.0 (7)	O4—C44—N8	124.9 (9)
N1—C1—C2	108.7 (7)	C49—C45—C44	120.8 (8)
N1—C1—C20	125.9 (7)	C46—C45—C44	121.0 (8)
C20—C1—C2	125.4 (7)	C46—C45—C49	118.1 (9)
N2—C19—C18	108.3 (6)	C45—C49—H49	118.3
N2—C19—C20	126.4 (7)	N12—C49—C45	123.4 (9)
C20—C19—C18	125.3 (7)	N12—C49—H49	118.3
N2—C16—C17	108.7 (6)	C45—C46—H46	120.3
N2—C16—C15	125.4 (7)	C47—C46—C45	119.5 (10)
C15—C16—C17	125.9 (7)	C47—C46—H46	120.3
N3—C14—C13	108.9 (6)	C46—C47—H47	120.6
N3—C14—C15	124.8 (7)	C48—C47—C46	118.8 (10)
C15—C14—C13	126.2 (7)	C48—C47—H47	120.6
N3—C11—C12	109.4 (7)	C47—C48—H48	118.0
N3—C11—C10	125.2 (7)	N12—C48—C47	123.9 (10)
C10—C11—C12	125.3 (7)	N12—C48—H48	118.0
N4—C9—C8	109.3 (6)	N7—C32—C33	115.2 (8)
N4—C9—C10	125.8 (7)	O2—C32—C33	121.0 (8)
C10—C9—C8	124.9 (7)	O2—C32—N7	123.7 (9)
N4—C6—C7	110.0 (7)	C34—C33—C32	119.7 (8)
N4—C6—C5	125.7 (7)	C37—C33—C32	120.8 (9)
C5—C6—C7	124.2 (7)	C37—C33—C34	119.3 (9)
C4—C3—H3	126.8	C33—C34—H34	119.6
C2—C3—C4	106.4 (7)	C35—C34—C33	120.8 (10)
C2—C3—H3	126.8	C35—C34—H34	119.6
C1—C2—H2	126.1	C33—C37—H37	119.8
C3—C2—C1	107.9 (7)	C33—C37—N13	120.4 (11)
C3—C2—H2	126.1	N13—C37—H37	119.8
C19—C18—H18	126.0	C34—C35—H35	121.0
C17—C18—C19	108.0 (7)	C34—C35—C36	118.0 (11)
C17—C18—H18	126.0	C36—C35—H35	121.0
C16—C17—H17	126.5	C35—C36—H36	118.1
C18—C17—C16	107.1 (7)	N13—C36—C35	123.7 (13)
C18—C17—H17	126.5	N13—C36—H36	118.1
C14—C13—H13	126.2	N10—C68—C69	115.8 (8)
C12—C13—C14	107.5 (7)	O3—C68—C69	121.5 (9)
C12—C13—H13	126.2	O3—C68—N10	122.6 (9)
C11—C12—H12	126.3	C73—C69—C68	121.4 (8)
C13—C12—C11	107.5 (7)	C70—C69—C68	120.3 (9)
C13—C12—H12	126.3	C70—C69—C73	118.2 (9)
C9—C8—H8	126.5	C69—C73—H73	118.2
C7—C8—C9	107.0 (7)	N11—C73—C69	123.6 (9)
C7—C8—H8	126.5	N11—C73—H73	118.2
C6—C7—H7	126.6	C69—C70—H70	120.9
C8—C7—C6	106.8 (7)	C71—C70—C69	118.2 (11)
C8—C7—H7	126.6	C71—C70—H70	120.9
C4—C5—C6	125.4 (7)	C70—C71—H71	119.8
C4—C5—C26	118.2 (7)	C70—C71—C72	120.5 (11)
C6—C5—C26	116.3 (7)	C72—C71—H71	119.8
C1—C20—C62	115.4 (7)	C71—C72—H72	119.2
C19—C20—C1	126.5 (7)	N11—C72—C71	121.6 (11)
C19—C20—C62	118.0 (7)	N11—C72—H72	119.2
C16—C15—C14	126.4 (7)	N9—C56—C57	114.5 (8)
C16—C15—C50	116.8 (7)	O1—C56—C57	121.2 (8)
C14—C15—C50	116.7 (7)	O1—C56—N9	124.2 (9)
C11—C10—C38	116.6 (7)	C58—C57—C56	121.6 (8)
C9—C10—C11	126.5 (7)	C61—C57—C56	120.1 (8)
C9—C10—C38	116.7 (7)	C61—C57—C58	118.2 (9)
C43—C38—C10	119.6 (7)	C57—C58—H58	120.2
C39—C38—C10	121.6 (8)	C59—C58—C57	119.6 (11)
C39—C38—C43	118.7 (7)	C59—C58—H58	120.2
C38—C43—N8	117.7 (7)	C57—C61—H61	117.9
C42—C43—C38	120.5 (8)	N14—C61—C57	124.1 (11)
C42—C43—N8	121.8 (8)	N14—C61—H61	117.9
C43—C42—H42	120.8	C58—C59—H59	120.8
C41—C42—C43	118.4 (9)	C60—C59—C58	118.4 (12)
C41—C42—H42	120.8	C60—C59—H59	120.8
C42—C41—H41	118.8	C59—C60—H60	117.0
C40—C41—C42	122.3 (8)	C59—C60—N14	126.0 (11)
C40—C41—H41	118.8	N14—C60—H60	117.0
C41—C40—H40	120.7	C5S—C1S—H1S	120.0
C41—C40—C39	118.7 (9)	C5S—C1S—C3S	120.0
C39—C40—H40	120.7	C3S—C1S—H1S	120.0
C38—C39—C40	121.3 (8)	C1S—C5S—H5S	120.0
C38—C39—H39	119.4	C1S—C5S—C4S	120.0
C40—C39—H39	119.4	C4S—C5S—H5S	120.0
C31—C26—C5	120.8 (7)	C5S—C4S—Cl1S	122.5 (7)
C27—C26—C5	121.1 (7)	C2S—C4S—C5S	120.0
C27—C26—C31	118.1 (7)	C2S—C4S—Cl1S	117.4 (7)
C26—C31—N7	118.5 (7)	C4S—C2S—H2S	120.0
C30—C31—C26	120.9 (8)	C4S—C2S—C6S	120.0
C30—C31—N7	120.6 (7)	C6S—C2S—H2S	120.0
C31—C30—H30	120.1	C2S—C6S—H6S	120.0
C29—C30—C31	119.8 (8)	C3S—C6S—C2S	120.0
C29—C30—H30	120.1	C3S—C6S—H6S	120.0
C30—C29—H29	119.7	C1S—C3S—H3S	120.0
C30—C29—C28	120.5 (8)	C6S—C3S—C1S	120.0
C28—C29—H29	119.7	C6S—C3S—H3S	120.0
C29—C28—H28	120.2	C8S—C7S—Cl2S	119.6 (9)
C27—C28—C29	119.7 (9)	C10S—C7S—C8S	121.9 (11)
C27—C28—H28	120.2	C10S—C7S—Cl2S	118.5 (10)
C26—C27—H27	119.6	C7S—C8S—H8S	120.1
C28—C27—C26	120.9 (8)	C9S—C8S—C7S	119.8 (11)
C28—C27—H27	119.6	C9S—C8S—H8S	120.1
C67—C62—C20	120.0 (7)	C8S—C9S—H9S	119.7
C63—C62—C20	120.6 (7)	C8S—C9S—C12S	120.7 (13)
C63—C62—C67	119.4 (7)	C12S—C9S—H9S	119.7
C62—C67—C66	120.1 (8)	C7S—C10S—H10S	120.9
C62—C67—N10	118.7 (7)	C7S—C10S—C11S	118.3 (11)
N10—C67—C66	121.2 (8)	C11S—C10S—H10S	120.9
C67—C66—H66	120.8	C10S—C11S—H11S	119.9
C65—C66—C67	118.4 (8)	C10S—C11S—C12S	120.2 (12)
C65—C66—H66	120.8	C12S—C11S—H11S	119.9
C66—C65—H65	119.0	C9S—C12S—C11S	118.9 (14)
C66—C65—C64	121.9 (8)	C9S—C12S—H12S	120.5
C64—C65—H65	119.0	C11S—C12S—H12S	120.5
C65—C64—H64	120.6	C21—N5—Mn1	125.6 (6)
C65—C64—C63	118.9 (8)	C24—N5—Mn1	127.6 (6)
C63—C64—H64	120.6	C24—N5—C21	106.6 (8)
C62—C63—C64	121.2 (8)	C22—N6—H6	125.8
C62—C63—H63	119.4	C24—N6—C22	108.3 (8)
C64—C63—H63	119.4	C24—N6—H6	125.8
C55—C50—C15	121.4 (7)	C31—N7—H7A	117.9
C51—C50—C15	120.6 (8)	C32—N7—C31	124.2 (7)
C51—C50—C55	118.0 (7)	C32—N7—H7A	117.9
C50—C55—N9	118.3 (7)	C43—N8—H8A	115.8
C54—C55—C50	120.7 (8)	C44—N8—C43	128.4 (7)
C54—C55—N9	121.0 (8)	C44—N8—H8A	115.8
C55—C54—H54	119.8	C55—N9—H9	117.3
C55—C54—C53	120.4 (9)	C56—N9—C55	125.4 (8)
C53—C54—H54	119.8	C56—N9—H9	117.3
C54—C53—H53	120.2	C67—N10—H10	114.7
C52—C53—C54	119.6 (8)	C68—N10—C67	130.6 (7)
C52—C53—H53	120.2	C68—N10—H10	114.7
C53—C52—H52	120.1	C72—N11—C73	117.8 (9)
C51—C52—C53	119.8 (9)	C49—N12—C48	116.2 (9)
C51—C52—H52	120.1	C36—N13—C37	117.4 (12)
C50—C51—H51	119.3	C61—N14—C60	113.5 (12)
C52—C51—C50	121.5 (8)		
			
Mn1—N1—C4—C3	163.1 (5)	C41—C40—C39—C38	2.0 (13)
Mn1—N1—C4—C5	−18.4 (11)	C39—C38—C43—C42	0.3 (12)
Mn1—N1—C1—C2	−163.2 (5)	C39—C38—C43—N8	179.7 (7)
Mn1—N1—C1—C20	18.9 (11)	C26—C31—C30—C29	0.5 (13)
Mn1—N2—C19—C18	164.8 (5)	C26—C31—N7—C32	118.2 (9)
Mn1—N2—C19—C20	−16.6 (11)	C31—C26—C27—C28	3.1 (12)
Mn1—N2—C16—C17	−163.8 (5)	C31—C30—C29—C28	0.8 (14)
Mn1—N2—C16—C15	17.8 (12)	C30—C31—N7—C32	−61.0 (12)
Mn1—N3—C14—C13	167.0 (5)	C30—C29—C28—C27	−0.1 (14)
Mn1—N3—C14—C15	−17.1 (12)	C29—C28—C27—C26	−1.9 (13)
Mn1—N3—C11—C12	−166.6 (6)	C27—C26—C31—C30	−2.5 (12)
Mn1—N3—C11—C10	17.3 (12)	C27—C26—C31—N7	178.4 (7)
Mn1—N4—C9—C8	169.2 (5)	C62—C67—C66—C65	0.3 (13)
Mn1—N4—C9—C10	−11.9 (11)	C62—C67—N10—C68	158.0 (9)
Mn1—N4—C6—C7	−168.4 (5)	C67—C62—C63—C64	3.5 (12)
Mn1—N4—C6—C5	16.3 (12)	C67—C66—C65—C64	0.4 (14)
N1—C4—C3—C2	0.8 (9)	C66—C67—N10—C68	−20.7 (14)
N1—C4—C5—C6	1.2 (14)	C66—C65—C64—C63	0.8 (13)
N1—C4—C5—C26	−175.0 (7)	C65—C64—C63—C62	−2.8 (13)
N1—C1—C2—C3	−1.3 (9)	C63—C62—C67—C66	−2.2 (12)
N1—C1—C20—C19	−2.0 (13)	C63—C62—C67—N10	179.2 (7)
N1—C1—C20—C62	173.4 (7)	C50—C55—C54—C53	−0.5 (13)
N2—C19—C18—C17	−0.3 (10)	C50—C55—N9—C56	128.5 (9)
N2—C19—C20—C1	0.8 (13)	C55—C50—C51—C52	2.6 (13)
N2—C19—C20—C62	−174.5 (7)	C55—C54—C53—C52	1.1 (14)
N2—C16—C17—C18	−1.5 (10)	C54—C55—N9—C56	−52.5 (13)
N2—C16—C15—C14	−1.2 (14)	C54—C53—C52—C51	0.1 (14)
N2—C16—C15—C50	175.9 (8)	C53—C52—C51—C50	−2.0 (13)
N3—C14—C13—C12	1.1 (10)	C51—C50—C55—C54	−1.3 (12)
N3—C14—C15—C16	0.7 (14)	C51—C50—C55—N9	177.7 (7)
N3—C14—C15—C50	−176.3 (7)	C21—C22—N6—C24	2.2 (12)
N3—C11—C12—C13	−2.2 (10)	C22—C21—N5—Mn1	−174.5 (7)
N3—C11—C10—C9	−4.3 (14)	C22—C21—N5—C24	0.2 (12)
N3—C11—C10—C38	170.1 (8)	C23—C22—N6—C24	−169.7 (13)
N4—C9—C8—C7	0.1 (9)	C25—C24—N5—Mn1	−5.7 (12)
N4—C9—C10—C11	1.3 (14)	C25—C24—N5—C21	179.7 (9)
N4—C9—C10—C38	−173.1 (7)	C25—C24—N6—C22	179.3 (9)
N4—C6—C7—C8	−2.4 (10)	C44—C45—C49—N12	177.9 (9)
N4—C6—C5—C4	−0.1 (14)	C44—C45—C46—C47	−179.8 (11)
N4—C6—C5—C26	176.2 (8)	C45—C44—N8—C43	−168.8 (8)
C4—N1—C1—C2	1.8 (9)	C45—C49—N12—C48	1.5 (15)
C4—N1—C1—C20	−176.1 (8)	C45—C46—C47—C48	2 (2)
C4—C3—C2—C1	0.3 (9)	C49—C45—C46—C47	−3.7 (17)
C4—C5—C26—C31	105.8 (9)	C46—C45—C49—N12	1.7 (15)
C4—C5—C26—C27	−73.7 (10)	C46—C47—C48—N12	1 (2)
C1—N1—C4—C3	−1.6 (9)	C47—C48—N12—C49	−2.9 (18)
C1—N1—C4—C5	176.8 (8)	C32—C33—C34—C35	177.9 (12)
C1—C20—C62—C67	−68.3 (10)	C32—C33—C37—N13	178.1 (13)
C1—C20—C62—C63	111.7 (9)	C33—C32—N7—C31	−163.5 (8)
C19—N2—C16—C17	1.3 (9)	C33—C34—C35—C36	0 (2)
C19—N2—C16—C15	−177.2 (8)	C33—C37—N13—C36	8 (3)
C19—C18—C17—C16	1.0 (10)	C34—C33—C37—N13	−7 (2)
C19—C20—C62—C67	107.5 (9)	C34—C35—C36—N13	0 (3)
C19—C20—C62—C63	−72.5 (10)	C37—C33—C34—C35	3 (2)
C16—N2—C19—C18	−0.6 (9)	C35—C36—N13—C37	−4 (3)
C16—N2—C19—C20	178.1 (8)	C68—C69—C73—N11	174.6 (9)
C16—C15—C50—C55	−65.6 (10)	C68—C69—C70—C71	−177.9 (12)
C16—C15—C50—C51	114.0 (9)	C69—C68—N10—C67	−165.4 (9)
C14—N3—C11—C12	2.9 (9)	C69—C73—N11—C72	3.2 (15)
C14—N3—C11—C10	−173.1 (8)	C69—C70—C71—C72	3 (2)
C14—C13—C12—C11	0.7 (10)	C73—C69—C70—C71	−2.0 (18)
C14—C15—C50—C55	111.7 (9)	C70—C69—C73—N11	−1.2 (15)
C14—C15—C50—C51	−68.7 (10)	C70—C71—C72—N11	−1 (2)
C11—N3—C14—C13	−2.5 (9)	C71—C72—N11—C73	−2.0 (17)
C11—N3—C14—C15	173.4 (8)	C56—C57—C58—C59	175.9 (11)
C11—C10—C38—C43	−68.7 (10)	C56—C57—C61—N14	179.8 (12)
C11—C10—C38—C39	113.0 (9)	C57—C56—N9—C55	−164.5 (8)
C9—N4—C6—C7	2.4 (9)	C57—C58—C59—C60	2 (2)
C9—N4—C6—C5	−172.8 (8)	C57—C61—N14—C60	6 (2)
C9—C8—C7—C6	1.3 (10)	C58—C57—C61—N14	−3.9 (18)
C9—C10—C38—C43	106.3 (9)	C58—C59—C60—N14	1 (3)
C9—C10—C38—C39	−72.0 (10)	C61—C57—C58—C59	−0.4 (17)
C6—N4—C9—C8	−1.6 (9)	C59—C60—N14—C61	−5 (3)
C6—N4—C9—C10	177.4 (8)	C1S—C5S—C4S—C2S	0.0
C6—C5—C26—C31	−70.7 (10)	C1S—C5S—C4S—Cl1S	177.2 (8)
C6—C5—C26—C27	109.7 (9)	C5S—C1S—C3S—C6S	0.0
C3—C4—C5—C6	179.4 (8)	C5S—C4S—C2S—C6S	0.0
C3—C4—C5—C26	3.2 (12)	C4S—C2S—C6S—C3S	0.0
C2—C1—C20—C19	−179.6 (8)	C2S—C6S—C3S—C1S	0.0
C2—C1—C20—C62	−4.2 (12)	C3S—C1S—C5S—C4S	0.0
C18—C19—C20—C1	179.2 (8)	C7S—C8S—C9S—C12S	−5 (2)
C18—C19—C20—C62	3.9 (12)	C7S—C10S—C11S—C12S	2 (2)
C17—C16—C15—C14	−179.3 (8)	C8S—C7S—C10S—C11S	−3.5 (18)
C17—C16—C15—C50	−2.3 (13)	C8S—C9S—C12S—C11S	3 (3)
C13—C14—C15—C16	175.9 (8)	C10S—C7S—C8S—C9S	4.8 (17)
C13—C14—C15—C50	−1.1 (12)	C10S—C11S—C12S—C9S	−2 (3)
C12—C11—C10—C9	−179.7 (8)	Cl1S—C4S—C2S—C6S	−177.3 (7)
C12—C11—C10—C38	−5.3 (12)	Cl2S—C7S—C8S—C9S	−175.1 (10)
C8—C9—C10—C11	−179.9 (8)	Cl2S—C7S—C10S—C11S	176.5 (10)
C8—C9—C10—C38	5.7 (12)	N5—C21—C22—C23	169.6 (14)
C7—C6—C5—C4	−174.7 (8)	N5—C21—C22—N6	−1.5 (13)
C7—C6—C5—C26	1.6 (12)	N5—C24—N6—C22	−2.1 (10)
C5—C4—C3—C2	−177.7 (8)	N6—C24—N5—Mn1	175.8 (5)
C5—C6—C7—C8	172.9 (8)	N6—C24—N5—C21	1.2 (10)
C5—C26—C31—C30	178.0 (8)	N7—C31—C30—C29	179.6 (8)
C5—C26—C31—N7	−1.1 (11)	N7—C32—C33—C34	37.1 (14)
C5—C26—C27—C28	−177.3 (8)	N7—C32—C33—C37	−148.0 (11)
C20—C1—C2—C3	176.6 (8)	N8—C43—C42—C41	−179.0 (8)
C20—C19—C18—C17	−179.0 (8)	N8—C44—C45—C49	34.5 (13)
C20—C62—C67—C66	177.8 (8)	N8—C44—C45—C46	−149.5 (10)
C20—C62—C67—N10	−0.9 (11)	N9—C55—C54—C53	−179.5 (8)
C20—C62—C63—C64	−176.5 (8)	N9—C56—C57—C58	36.3 (13)
C15—C16—C17—C18	177.0 (8)	N9—C56—C57—C61	−147.5 (10)
C15—C14—C13—C12	−174.7 (8)	N10—C67—C66—C65	178.9 (8)
C15—C50—C55—C54	178.3 (8)	N10—C68—C69—C73	39.6 (13)
C15—C50—C55—N9	−2.7 (12)	N10—C68—C69—C70	−144.7 (11)
C15—C50—C51—C52	−177.0 (8)	O1—C56—C57—C58	−140.0 (10)
C10—C11—C12—C13	173.8 (8)	O1—C56—C57—C61	36.2 (14)
C10—C9—C8—C7	−178.8 (8)	O1—C56—N9—C55	11.7 (15)
C10—C38—C43—C42	−178.1 (8)	O2—C32—C33—C34	−139.0 (11)
C10—C38—C43—N8	1.3 (11)	O2—C32—C33—C37	35.9 (16)
C10—C38—C39—C40	176.8 (8)	O2—C32—N7—C31	12.6 (15)
C38—C43—C42—C41	0.4 (13)	O3—C68—C69—C73	−138.5 (12)
C38—C43—N8—C44	159.6 (9)	O3—C68—C69—C70	37.2 (17)
C43—C38—C39—C40	−1.6 (13)	O3—C68—N10—C67	12.7 (18)
C43—C42—C41—C40	0.0 (14)	O4—C44—C45—C49	−145.3 (11)
C42—C43—N8—C44	−21.0 (14)	O4—C44—C45—C46	30.7 (16)
C42—C41—C40—C39	−1.2 (14)	O4—C44—N8—C43	11.0 (17)

### (2,5-Dimethylimidazole){N,N',N'',N'''-[porphyrin-5,10,15,20-tetrayltetra(2,1-phenylene)]tetrakis(pyridine-3-carboxamide)}manganese(II) chlorobenzene disolvate Hydrogen-bond geometry (Å, º)

**Table:**

*D*—H···*A*	*D*—H	H···*A*	*D*···*A*	*D*—H···*A*
N6—H6···O2^i^	0.88	2.05	2.858 (11)	153
N7—H7*A*···N11	0.88	2.24	3.094 (11)	164
N9—H9···N12	0.88	2.17	3.009 (10)	159
C5*S*—H5*S*···N14^ii^	0.95	2.57	3.421 (16)	150
C36—H36···O3^ii^	0.95	2.35	2.99 (2)	124
C60—H60···O4^iii^	0.95	2.40	3.062 (18)	126

Symmetry codes: (i) −*x*+1, −*y*, *z*+1/2; (ii) −*x*+1/2, *y*+1/2, *z*−1/2; (iii) −*x*+1/2, *y*−1/2, *z*+1/2.
