# Supplementary material for: (2,5-Di­methyl­imidazole){N,N′,N′′,N′′′-[porphyrin-5,10,15,20-tetra­yltetra­(2,1-phenyl­ene)]tetra­kis(pyridine-3-carboxamide)}manganese(II) chloro­benzene disolvate
Source: IUCrdata. 2024 Jun 4;9(Pt 6):x240497. doi: 10.1107/S2414314624004978 (PMC11223682; doi:10.1107/S2414314624004978)
Supplement: Supplementary file 3 [file x-09-x240497-sup3.docx]

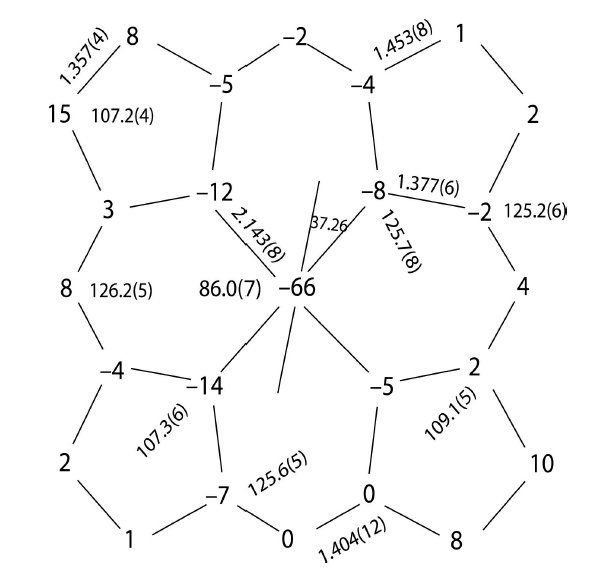


Supplementary Figure 1: a formal diagram of the porphyrinato core of the title compound. Averaged values of the chemically unique bond distances (in Å) and angles (°) are shown. The numbers in parentheses are the e.s.d.'s calculated on the assumption that the averaged values were all drawn from the same population. The perpendicular displacements (in units of 0.01Å) of the porphyrin core atoms from the 24-atom mean plane are also displayed. Positive values of the displacements are towards the hindered porphyrin side.
